# Exploration of Maternal and Fetal Toxicity Risks for Metronidazole-Related Teratogenicity and Hepatotoxicity through an Assessment in Albino Rats

**DOI:** 10.3390/toxics11040303

**Published:** 2023-03-25

**Authors:** Mervat A. AbdRabou, Barakat M. Alrashdi, Hadeel K. Alruwaili, Reda H. Elmazoudy, Maha A. Alwaili, Sarah I. Othman, Fawzyah A. Alghamdi, Gehan H. Fahmy

**Affiliations:** 1 Biology Department, College of Science, Jouf University, P.O. Box 2014, Sakaka 72388, Saudi Arabia; 2 Biology Department, College of Science, Imam Abdulrahman Bin Faisal University, P.O. Box 1982, Dammam 31441, Saudi Arabia; 3 Biology Department, College of Science, Princess Nourah Bint Abdulrahman University, Riyadh 11564, Saudi Arabia; 4 Biology Department, College of Science, University of Jeddah, Jeddah 23218, Saudi Arabia; 5 Biology Department, College of Science, Taibah University, Al-Madinah Al-Munawwarah 30001, Saudi Arabia

**Keywords:** metronidazole, pregnancy, embryos, preterm birth, birth defects, placenta

## Abstract

Metronidazole is the primary antimicrobial drug for treating acute and chronic vaginal pathogens during pregnancy; however, there has been insufficient research on placental disorders, early pregnancy loss, and preterm birth. Here, the potential activity of metronidazole on pregnancy outcomes was investigated. 130 mg/kg body weight of metronidazole was orally given individually to pregnant rats on gestation days 0–7, 7–14, and 0–20. Pregnancy outcome evaluations were carried out on gestation day 20. It was demonstrated that metronidazole could induce maternal and fetal hepatotoxicity. There is a significant increase in the activities of maternal hepatic enzymes (ALT, AST, and ALP), total cholesterol, and triglycerides compared with the control. These biochemical findings were evidenced by maternal and fetal liver histopathological alterations. Furthermore, metronidazole caused a significant decrease in the number of implantation sites and fetal viability, whereas it caused an increase in fetal lethality and the number of fetal resorptions. In addition, a significant decrease in fetal weight, placental weight, and placental diameter was estimated. Macroscopical examination revealed placental discoloration and hypotrophy in the labyrinth zone and the degeneration of the basal zone. The fetal defects are related to exencephaly, visceral hernias, and tail defects. These findings suggest that the administration of metroniazole during gestation interferes with embryonic implantation and fetal organogenesis and enhances placental pathology. We can also conclude that metronidazole has potential maternal and fetal risks and is unsafe during pregnancy. Additionally, it should be strictly advised and prescribed, and further consideration should be given to the associated health risks.

## 1. Introduction

Females are among the most vulnerable to being infected with vaginitis caused by parasites or pathologies related to childbirth and the care of women giving birth [1]. Intentionally or unintentionally, women rely broadly on medication or pharmaceutical therapy to treat protozoal and/or bacterial infections during pregnancy or when not pregnant [2].

Pregnancy evaluation focuses on the maternal and/or fetal implications of drug use. Furthermore, embryos/fetuses are more susceptible in pregnant females treated with medications [3]. Perinatal exposure to drugs leads to abnormal intrauterine embryo/fetus development manifested by growth delay, organ deterioration, and fetal resorption or death [4]. These drugs can penetrate the placental membrane’s maternal-fetal barrier and disrupt normal fetal development [5].

Metronidazole is an antibiotic drug synthesized by actinobacteria and proteobacterial genera and is used to cure Bacteroides infections and certain parasitic illnesses [6]. It has been an effective prescribed medication against human vaginitis infections in gynecology and obstetrics such as *Trichomonas vaginalis*, *Entamoeba histolytica*, and *Giardia lamblia* for many years. The metronidazole-available doses are injectable, intravenous, vaginally, and rectally [7].

Various literature reports that metronidazole has therapeutic effects; however, its safety during pregnancy has not been fully confirmed [8,9]. Concerns have been raised about the potential side effects of treating pregnant women with metronidazole [10]. According to the Food and Drug Administration (FDA), metronidazole is classified as having a category B risk for damaging fetuses, but its use still provokes divided opinion among physicians. [11]. In this classification, harmful action is evident in the first trimester of the gestation period [12]. The majority do not advocate it during the first trimester, while in the second and third trimesters it is justified only in cases where alternative therapy is unsuccessful [13].

Toxicological studies demonstrated that metronidazole is bioavailable and can be distributed in body fluids [14] and extend across the maternal-fetal barrier into the embryo/fetus circulation and amniotic fluid [15]. Through this potential effect, developmental retardation, deformed organs, and fetal death can be observed. Furthermore, metronidazole administration can directly influence fetogenesis independent of maternal toxicity [16].

Metronidazole is considered to have broad toxicological prospects compared to most xenobiotics due to its biotransformation in the liver through oxidation, hydroxylation, and conjugation of metronidazole glucuronide [17]. Moreover, a cumulative number of studies on animals and humans indicated an association of metronidazole with the disturbance of alanine aminotransaminase (ALT), aspartate aminotransaminase (AST), alkaline phosphatase (ALP), total cholesterol (TC), and triglyceride (TG), which are factors involved in hepatotoxicity [18]. Metronidazole also enhances steatosis-related early-stage hepatocarcinogenesis and induces liver tumors through increased hepatic neoplasms [19].

Experimental research showed that metronidazole can induce post-implantation embryo lethality in rats [20]. Others declared a probable relationship between the administration of vaginal metronidazole and hydrocephaly during pregnancy [21]. In addition, Shennan et al. [22] reported that metronidazole therapy might increase the chance of preterm birth and/or abortions during pregnancy.

Previous published meta-analyses answered whether metronidazole exposure during the first trimester of pregnancy is associated with an increased teratogenic risk in humans. The outcome under consideration was the occurrence of birth defects in live-born infants. It was concluded that metronidazole does not appear to be associated with an increased teratogenic risk [23]. On the other hand, other meta-analysis data do not confirm the efficacy of metronidazole in reducing the risk of preterm birth and associated delivery outcomes [24]. Hence, further research is required to confirm the effect of high doses and a short duration of metronidazole treatment on preterm birth among the high-risk group.

Consequently, in addition to maternal toxicity, it is evident that metronidazole has the potential to disrupt the normal intrauterine development of the embryo/fetus. So, the present study and the body of literature were planned to investigate methodically the effect of metronidazole during different trimesters and the whole pregnancy on fetal development in pregnant rats.

## 2. Materials and Methods

### 2.1. Metronidazole Drug

Metronidazole (Flagyl^®^, 500 mg per capsule) was purchased from Sanofi global biopharmaceutical company, a Saudi Arabian branch (Jouf, Saudi Arabian). The dose prepared for the current study was calculated (130 mg/kg) according to [2] and was dissolved in a physiological saline solution. 

### 2.2. Animal Care and Use 

All procedures of the present study were carried out in compliance with standards, rules of Institutional Ethics Use and the Care Committee of Laboratory Animals at Jouf University. This study was conducted on sexually mature and experimentally naïve female rats weighing about 160–180 g at the beginning of the experiment. Female rats were kept in cages with free access to conventional drinking water and food at 25 ± 2 °C. The relative humidity levels were maintained at 50 ± 5%.

### 2.3. Determination of the Estrous Cycle 

Every morning, vaginal smears were obtained for vaginal cytology to identify the phases of the oestrous cycle. Proestrus is characterized by a predominance of nucleated epithelial cells and a small proportion of cornified epithelial cells. During the estrus phase, cornified squamous epithelial cells will predominate. Leukocytes and cornified squamous epithelial cells are present during the metestrus phase. In the diestrus phase, leukocytes will be the predominant cell type [25].

### 2.4. Mating and Pregnancy Snippets

After two weeks of adaptation, females with three consecutive regular oestrous cycles were caged with a proven fertile male (2 females: 1 male). The next morning, to check if sperm were present, vaginal smears were taken. The appearance of spermatozoa in vaginal smears or the existence of a vaginal plug was regarded as the onset of gestation [26].

### 2.5. Experimental Design

The timed pregnant females were arbitrarily divided into four groups, (10 females/each). The control group (GC): pregnant rats received 0.5 mL of physiological saline solution from day zero through the twentieth day of gestation. The first experimental group (GMI): pregnant rats treated with metronidazole from day zero through the seventh day of gestation. The second experimental group (GMII): pregnant rats received metronidazole from day zero through the fourteenth day of pregnancy. The third experimental group (GMIII): pregnant females received metronidazole from day zero through the twentieth day of gestation. Metronidazole was given orally by gavage at a daily dosage of 130 mg/kg body weight in 0.5 mL of physiological saline solution after confirmation of mating.

### 2.6. Narrative Toxicological Signs of Dams

The perinatal screening of pregnant female rats was reviewed to determine whether metronidazole treatment induces vaginal bleeding. In addition, a gross analysis of the maternal toxicity of pregnant females from all groups was monitored throughout the pregnancy trimesters. Preterm births, spontaneous abortions, and maternal deaths before the 20th day of gestation (via cesarean section) were also screened during the period of pregnancy.

## 3. Pregnancy Outcomes Evaluation

### 3.1. Dissection Procedure of Pregnant Rats

On the 20th day of gestation (9:00 a.m.), all pregnant rats from each group were intramuscularly anesthetized with (80 mg/kg ketamine hydrochloride and 6 mg/kg xylazine intramuscularly) and then euthanized and dissected under a dissecting stereomicroscope. Dissection procedures are performed on a black background and performed as quickly as possible to maintain the viability of the samples. After a transverse incision of the abdomen with scissors, the gravid uterine horns were carefully exposed and excised with forceps by cutting at the cervix and along the mesometrium. At necropsy, the fetuses were pulled out, aseptically detached, and removed from the uterus with the attached and intact placenta and yolk sac. The placenta discs were secluded from the uterine wall. After that, the isolated placenta and fetuses were externally washed with 70% ethanol, air-dried, and individually weighed. The uterine horns were inspected to estimate the number and location of implantation sites, fetal resorptions (early or late), fetal death, and viable fetuses. Fetal viability was evaluated by the presence or absence of fetal size, fetal movement, skin coloration, and fetal developmental stage. The uteri of females that cannot be easily seen or noticed to be pregnant can be inspected by staining the uterine horns with 10% ammonium sulfide [27].

### 3.2. Macroscopical Evaluation

#### 3.2.1. Fetus and Placenta 

The crown-rump length of each fetus was measured. Thereafter, the collected fetuses were inspected for the skull, eyes, ears, limbs, and tail to carry out a possible systematic inquiry and identify the existence of the malformations of external morphology. Macroscopically, at necropsy, the placenta was also individually evaluated for abnormal or distinctive attributes or aspects in anatomy and pathology, and an image was acquired using a dissecting stereomicroscope.

#### 3.2.2. Blood and Serum Biochemistry

The blood samples were placed in a 1.5 mL anticoagulation centrifuge tube for one hour and then centrifuged at 4 °C and 10,000× *g* rpm for 10 min to obtain the serum. Alanine aminotransferase (ALT), aspartate aminotransferase (AST), alkaline phosphatase (ALP), total protein (TP), total cholesterol (TC), triglyceride (TG), high-density lipoprotein cholesterol (HDL-C), and low-density lipoprotein cholesterol (LDL-C) assay kits (Mindray, Shenzhen, China) were removed from the refrigerator at 4 °C and tested by a fully automated biochemical analyzer.

#### 3.2.3. Histopathological Analysis

The largest lobe of the weighed liver was soaked in 4% paraformaldehyde, dehydrated with a certain gradient of ethanol, embedded in paraffin, and stained with hematoxylin-eosin (HE) at a thickness of 4 μm. Microscopy examined histopathology to assess the extent of liver injury in all sections [28].

## 4. Statistical Analysis

Statistical data analysis package for science (Origin 2019b SPSS—version 23) for Windows was applied. To analyze differences among all treatments, the one-way analysis of variance (ANOVA) is followed by Duncan’s test for the difference between groups. The data were expressed as the mean and standard deviation (mean ± standard deviations (SDs)) at *p* ≤ 0.05.

## 5. Results

### 5.1. Maternal Toxicity

Observational findings evaluated the safety of metronidazole administration, and the statistical analysis showed no significant results during the three trimesters. Pregnant rats of the three groups (GC, GMI, GMII, and GMIII) did not display any evident symptoms of maternal toxicity or undesirable behavior. No maternal mortality or morbidity was noticed among the pregnant rats during the pregnancy period. Regarding vaginal bleeding, most pregnant rats did not exhibit it, starting around GD 0–7th, 7–14th, or 0–20th days. Furthermore, no clinical signs of hemorrhage were observed in gravid uteri in treated females at necropsy compared with controls. No changes in skin and fur, eyes, and mucous membranes, respiratory and digestive distress, behavior patterns, or coma were observed in the pregnant females. Furthermore, no gross pathological changes were monitored at necropsy in the tissues and organs of surviving rats.

### 5.2. Preterm Birth and Abortion 

Statistically, there were no preterm births among all pregnant female rats treated with metronidazole in any trimester compared to pregnant female rats without metronidazole treatment. Furthermore, no significant abortion was observed among metronidazole-treated pregnant female rats. 

### 5.3. Maternal Body Weight

Excluding the dead rats, the females in the experimental and control groups showed a steady body weight during the days of the experimental period. 

### 5.4. Liver Coefficients

**Figure 1:**
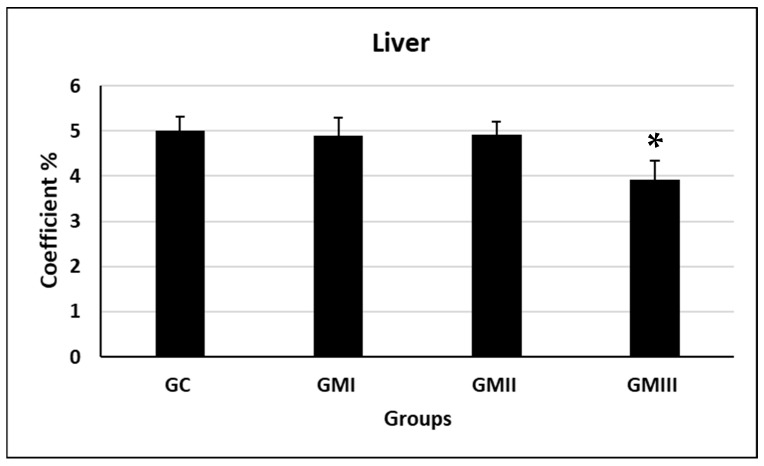
Liver coefficient of pregnant rats (*n* = 10.) * Significant differences with respect to the control group (GC) *p* ≤ 0.05.

Compared with the control group, the differences in liver coefficients in the GI and GII experimental groups of rats were not statistically significant, while the difference in liver coefficients in the GIII experimental group was significantly decreased (*p* ≤ 0.05, Figure 1).

## 6. Indices of Pregnancy Outcomes

### 6.1. Gross Gravid Uterine Horns

**Figure 2:**
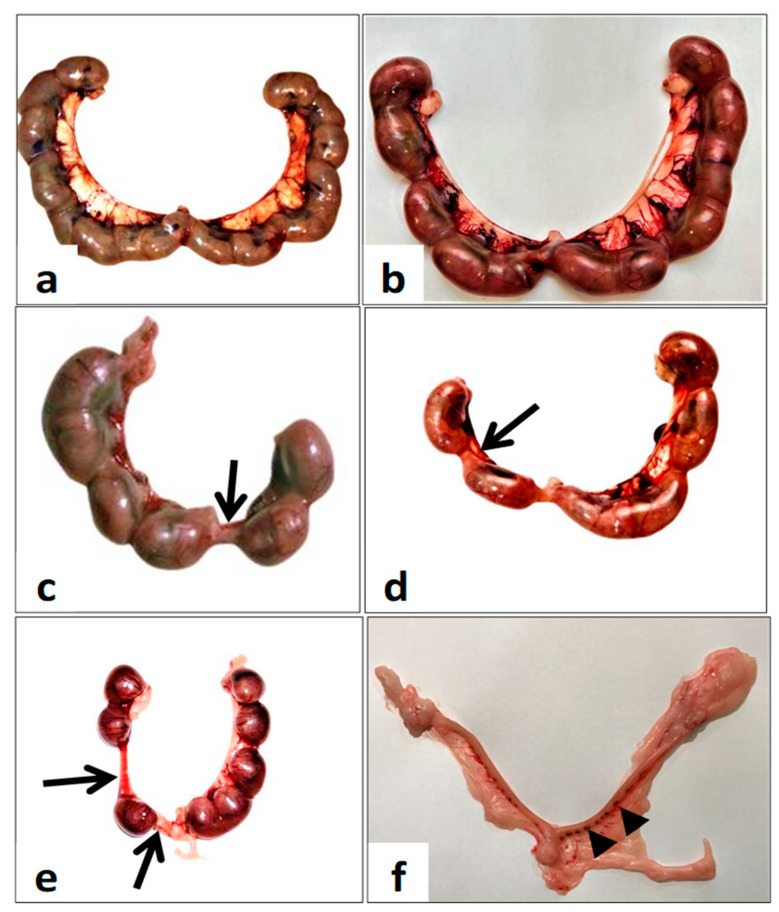
The whole mount of gravid uterine horns from pregnant rats was sacrificed on the 20th day of gestation. (**a**,**b**) The control group with full-term fetuses. (**c**) group I (day 0–7) show missed and resorbed fetuses (arrows). (**d**) Group II (day 7–14) shows resorbed fetuses (arrows) and intrauterine growth retardation of fetuses (arrows). (**e**) Group III (day 0–20) show resorbed fetuses (arrows) and dead and retarded fetuses. (**f**) Postimplantation loss (arrowheads).

Each gravid uterine horn contains multiple healthy fetuses, each within its own separate yolk sac and attached to the uterus via the umbilical cord and a discoid placenta (Figure 2a,b). The number of implanted fetuses per gravid uterine horn decreased compared with the control group. On the other hand, there was an increase in resorbed fetuses in the uterine horns of females in groups I and II (Arrow, Figure 2c,d) and group III (Arrow, Figure 2e). Furthermore, group III uterine horns suffered from complete fetal resorption and appeared as resorbed implantation sites (Arrowhead, Figure 2f).

### 6.2. Day 0–7 Experiment Findings

In this group, there was a significant decrease in both the number of implantation sites and the number of viable fetuses compared to the control group (*p* ≤ 0.05, Table 1). On the other hand, the number of resorption sites was significantly increased compared with the control group. The fetal body weight and crown-rump length were not significantly affected in this treated group compared to the ad libitum control group (*p* ≤ 0.05, Table 1). No significant difference was observed in the number of dead fetuses in metronidazole-treated rats compared to the control group (*p* ≤ 0.05, Table 1).

### 6.3. Day 7–14 Experiment Findings

The statistical analysis of pregnancy outcomes showed a significant decrease in both the number of implantation sites and the number of viable fetuses compared to the control group (*p* ≤ 0.05, Table 1). While the number of resorptions and dead fetuses significantly increased compared to the control group (*p* ≤ 0.05, Table 1). Concerning the fetal growth parameters, metronidazole produced a significant reduction in fetal body weight, and crown-rump length (*p* ≤ 0.05, Table 1). 

### 6.4. Day 0–20 Experiment Findings

The results of the whole pregnancy period (day 0–20) treatment with metronidazole showed a significant decrease in the mean number of implantation sites and the number of live fetuses (*p* ≤ 0.05, Table 1) compared to the control group. A significant decrease in fetal body weight and fetal crown-rump length was observed compared to the control group (*p* ≤ 0.05, Table 1). The total number of fetuses per group was significantly decreased in all treated groups during the pregnancy trimesters compared to the corresponding control group (*p* ≤ 0.05, Table 1). 

### 6.5. Placenta Weight and Diameter

**Figure 3:**
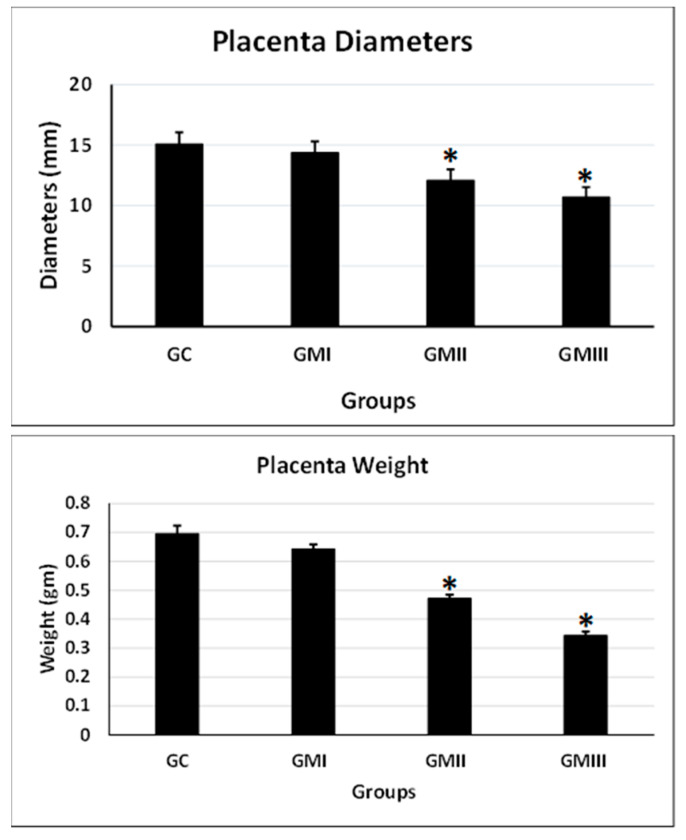
Evaluation of placental parameters (weights and diameters) in the treated pregnant rats with metronidazole. Values are expressed as mean ± standard deviation (M ± SD) *n* = 10/group. * The values are significantly different at *p* ≤ 0.05 (ANOVA) with Duncan’s multiple range test.

Compared with the control group, the differences in placenta weight and diameter in the GII and GIII experimental groups of rats were significantly decreased compared with the control group (*p* ≤ 0.05, Figure 3). While no significant difference was observed in the GI compared with the control group (*p* ≤ 0.05, Figure 3). 

### 6.6. Placental Morphology and Anatomy

**Figure 4:**
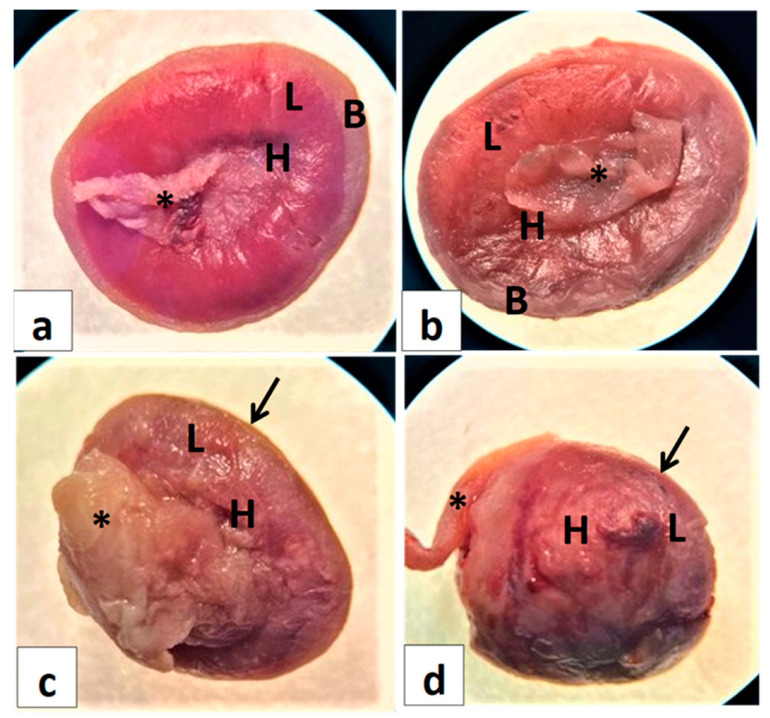
Photomacrographs of the frontal view of placental morphologies in control and pregnant rats treated with metronidazole at different gestation periods. (**a**) Normal control, red-colored labyrinth (L), thick basal decidua (B). (**b**) Group I indicate discoloration of the labyrinth (L) and adherence of the umbilical cord (*) to the chorionic surface (H). (**c**) Group II indicates placental hypotrophy, and thin basal decidua (arrow). (**d**) Group III indicates reduced placenta and placental hypotrophy, necrosis, and the disappearance of basal decidua (arrow).

Normally, in the frontal view, the fetal surface of the placenta facing the fetus wherein enters the umbilicus appears as a dark red zone due to the high vascularization of the labyrinth (L, Figure 4a), called the chorionic plate (H, Figure 4a). The adjacent zone appears yellow, representing the junctional zone. The fetal surface placenta comprises the fetus and is composed of three compartments, encompassing the yolk sac, chorionic plate, labyrinth, and junctional zone. While the maternal surface includes the decidua and is called the basal plate (Arrow, Figure 4a). As shown in Figure 4a and compared with the normal rat placentas, the GI placenta showed normal architecture with three compartments: the chorionic zone (H, Figure 4b), the labyrinth zone (L, Figure 4b), and the maternal decidua basalis (Arrow, Figure 4b). However, the labyrinth zone shows discoloration and adherence of the umbilical cord to the chorionic surface (L, Figure 4b). The GII placentas exhibited hypotrophy with disrupted structures such as the labyrinth (L, Figure 4c) and decreased thickness of the decidual basal layer that lacked differentiation (Arrow, Figure 4c). On the other hand, the placentas in the GIII appeared more hypotrophic with decreased diameter and thickness compared with control placentas (Figure 4d). In addition, the degeneration of the decidual basal layer (Arrow, Figure 4d).

### 6.7. Gross Morphology of Fetal Abnormalities

#### 6.7.1. Fetal Growth

**Figure 5:**
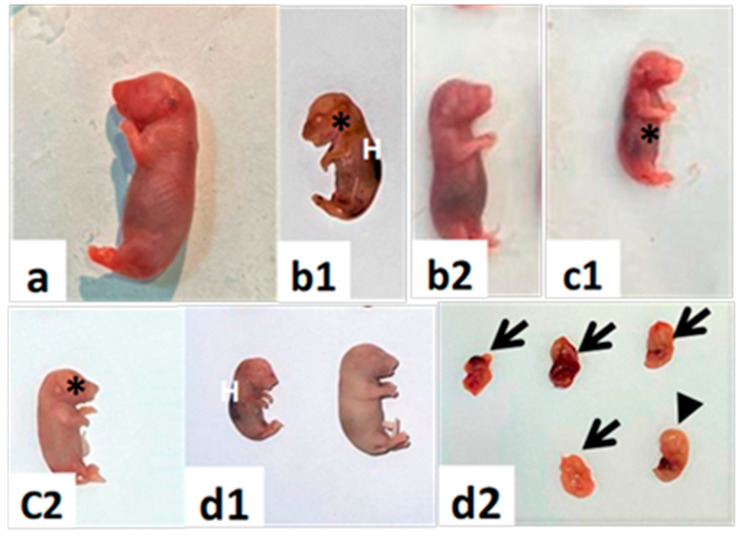
Photomacrographs of the whole mount of twentieth-day-old rat fetuses of pregnant rats treated with metronidazole at different gestation periods showing various morphological intrauterine growth restrictions (IUGR). (**a**) In the control group, the fetuses appeared healthy and normal. (**b1**, **b2**) Group I, (**c1**, **c2**) Group II, and (**d1**, **d2**) Group III. Notice growth retardation (*), dead fetus (arrowhead), partially resorbed fetuses (arrows), and hematomas (H).

Figure 5 demonstrates the gross morphological abnormalities of fetuses. These results showed that metronidazole resulted in intrauterine growth retardation or restriction at 20 days of gestation, indicating that metronidazole can be implicated in fetal development. The hematoma was recorded to occur more frequently among the treated groups compared with the control groups. 

#### 6.7.2. Major Congenital Anomalies

**Figure 6:**
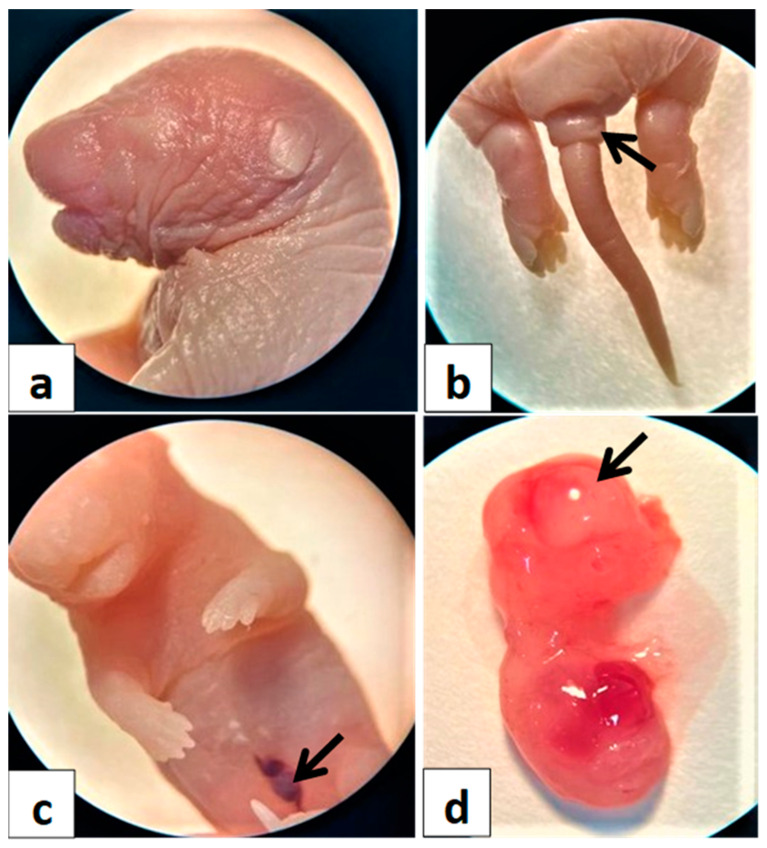
The major external and visceral morphological anomalies of pregnant rats treated with metronidazole during the 0–20th days of the gestational period. (**a**) control group, (**b**) show tail defects (arrow), (**c**) show visceral hernia (arrow), and (**d**) indicate exencephaly (arrow).

Congenital anomalies were observed more frequently in GMIII compared with the GMI, GMII, and GC control groups (Figure 6). Such that, treatment with metronidazole during the three trimesters (day 0–20) produced morphological anomalies in 20-day-old fetuses compared with control fetuses (Figure 6a). The major congenital malformations were exencephaly anomalies, visceral hernias, and tail defects (Figure 6b–d).

#### 6.7.3. Blood Chemistry

**Figure 7:**
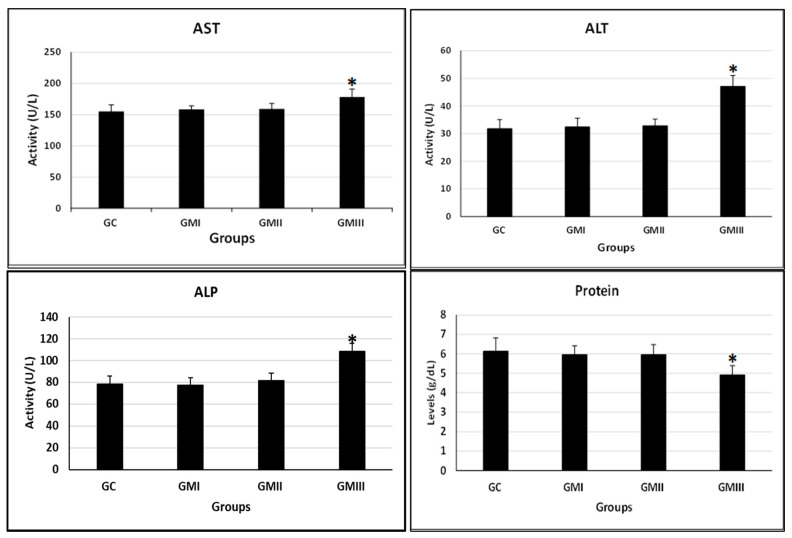
Effect of metronidazole on the serum liver function index of pregnant albino rats. Values are expressed as mean ± standard deviation (mean ± SD) *n* = 10/group. * The values are significantly different at *p* ≤ 0.05 (ANOVA) with Duncan’s multiple range test.

Compared with the control group, the GIII experimental group caused significant alterations in liver functions (*p* ≤ 0.05, Figure 7). The GIII group caused a significant increase in levels of ALT, AST, ALP, and total protein compared with the control group (*p* ≤ 0.05, Figure 7). Furthermore, compared with the control group, triglycerides (TG, lipid index), total cholesterol, and HDL-C only increased in the GIII group (*p* ≤ 0.05, Table 2). There is no significant alteration in the activity of LDL-C compared with the control group (*p* ≤ 0.05, Table 2).

### 6.8. Hepatic Histopathology

#### 6.8.1. Maternal Hepatotoxicity 

**Figure 8:**
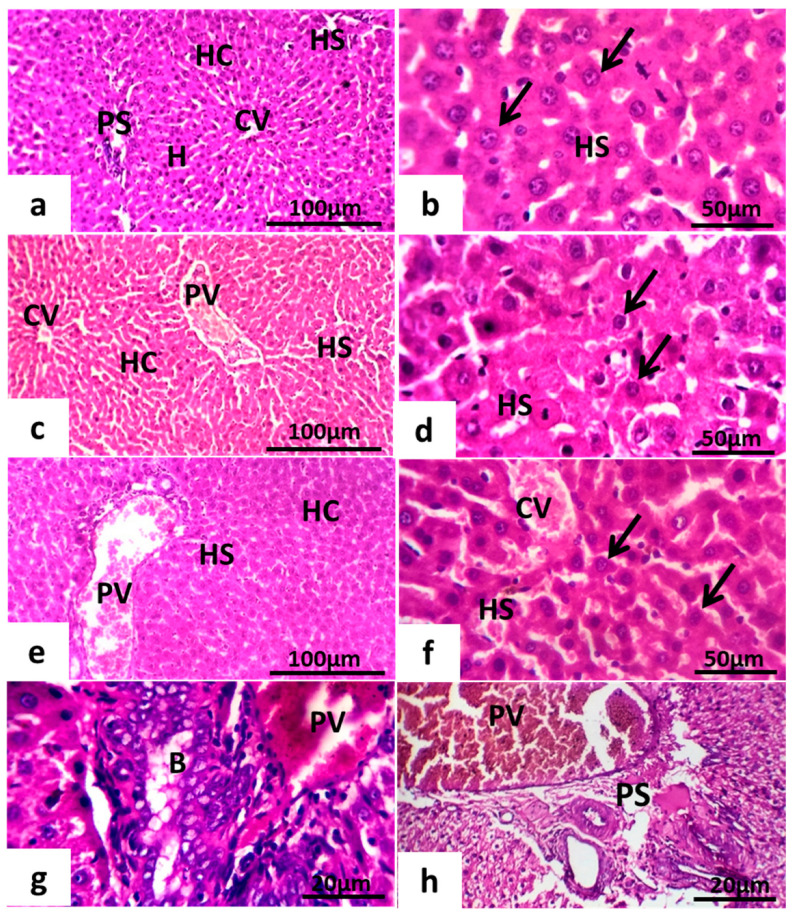
Photomicrograph of maternal rats’ liver. (**a**,**b**) Normal control group. (**c**,**d**) treated females at (0–7th dpc). (**e**,**f**) treated female at (7–14th dpc). (**g**,**h**) treated female at (0–20th dpc). Notice the portal space (PS), portal vein (PV), central vein (CV), hepatocytes (HC, arrows) of hepatic strands (H), and hepatic sinusoids (HS). H&E.

Histological examination illustrates that the maternal liver displays a normal architecture of hepatocytes with a distinct mitotic index in the liver (Figure 8a). The histological analysis of the maternal liver showed different degrees of histopathological alterations in all experimental groups compared with the control group (Figure 8). In contrast to the control group, increased severity of ground parenchyma was observed in the GII and GIII groups (Figure 8e–h). The liver in the GII group showed mild inflammatory cell infiltrates and blood vessel congestion (Figure 8e,f). The liver tissue of rats in the GIII group showed the disappearance of the hepatic cord, swollen hepatocytes, broken cells, and pyknosis of hepatocytic nuclei (Figure 8g,h). Compared with the control group, the GI group had less liver damage (Figure 8c,d).

#### 6.8.2. Fetal Hepatotoxicity

**Figure 9:**
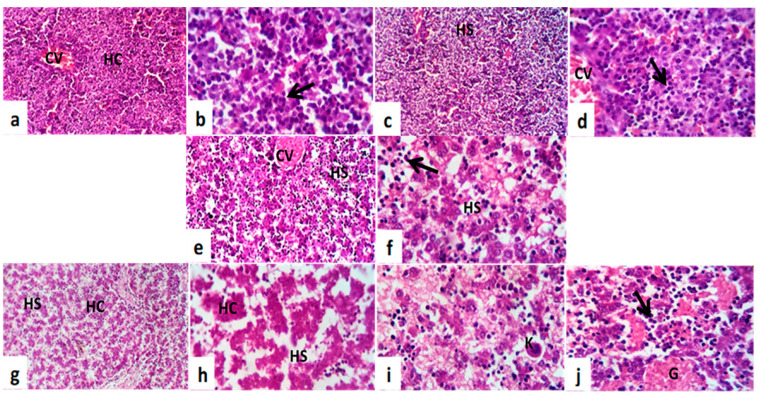
Photomicrographs of fetal liver tissue sections of metronidazole-maternally treated mice. (**a**,**b**) Normal control group, (**c**,**d**) treated female at (0–7th dpc), (**e**,**f**) treated female at (7–14th dpc), and (**g**–**j**) treated female at (0–20th dpc). Notice the central vein (CV), hepatic cords (HC), hepatic sinusoids (HS), circulate nucleated erythrocytes, mature hemopoietic cells (arrows), megakaryocytes (K), and hemorrhage (G). H&E. ((**a**,**c**,**e**,g) = Scale 100 μm) ((**b**,**d**,**f**,**h**–**j**) = Scale 50 μm).

Histological examination illustrates that the normal fetal liver is mostly comprised of hepatic cords and sinusoids at this stage of development. The hepatic cords are composed of primarily and largely undifferentiated hepatoblasts (Figure 9a,b). RBCs are found within the vessels. Most of the hematopoietic cell population is of the erythroid lineage and can be identified by the intense, hyperchromatic nuclei (Figure 9a,b). The architecture of the liver in the GI tract is nearly identical to that seen in the normal fetal liver (Figure 9c,d). Whereas GII showed disruptions in the hepatic organization of hepatocytes, defective hepatocyte maturation, and abnormal hepatic cord arrangements (Figure 9e,f). In the liver tissue sections of GIII, hepatocytes are small, round, and loose and associated with disruptions in hepatic architecture and cell morphology observable on stained liver tissue sections (Figure 9g,h). The liver parenchyma appears looser and less organized. In addition, megakaryocytes are present (Figure 9g,h).

## 7. Discussion

Metronidazole is the primary antimicrobial drug for treating acute and chronic vaginal pathogens during the gestation period; however, the limited literature on placental disorders and pregnancy outcomes has not been as sufficient as required. Furthermore, the placenta is crucial for fetal development and pregnancy success. So, in this study, we have attempted to confirm the in-utero effects of metronidazole given to pregnant rats on the observable disorders of the placenta along with pregnancy outcomes. 

The findings showed that metronidazole induced discernible lesions in the placenta and had detrimental effects on the consequences of conception disturbances in the normal gestational consistency. The reduction in the number of live fetuses observed in the present results was consistent with several reports. Consequently, there is a relationship between metronidazole administration during pregnancy and low fetal weight, the number of implantation sites, fetal viability, and congenital anomalies among the outcomes of pregnant female rats.

The crown-rump length, embryonic resorption or death, number of implantation sites, and embryonic morphology are indicators of great significance in reproductive toxicology during the embryonic development [29]. In pregnant rats of GMI, GMII, and GMIII, a decrease in the number of live fetuses and a reduction in the implantation sites may indicate that metronidazole is implicated in the pre-implantation and/or post-implantation processes [2]. In addition, a decrease in fetal viability may be attributed to the expelling effect of metronidazole on the blastocyst after fertilization or its potential cytotoxicity on oocyte liberation [30]. The evidence indicates that metronidazole can interfere with morphogenic pathways, inducing malformations and developmental toxicity such as adduction and transversions of GC-CG DNA [31]. It is also worth noting that metronidazole disrupts apoptosis and the proliferation of cell migration and maturation, causing embryonic defects [21]. Moreover, several studies point out that perinatal exposure to metronidazole increases intrauterine fetal growth restriction and malformation occurrence, referring to its mutagenic and teratogenic potency [32]. In this context, fetal resorption, death, and teratogenicity malformations could be strictly explained by the cytotoxicity and/or genotoxicity of metronidazole, which is in agreement with its suggestion of its ability to induce genotoxic effects on embryonic cells [33]. 

According to Talapatra et al. [34], metronidazole induces micronucleus and binucleus formation and increases the number of chromosome aberrations due to its genotoxic, cytogenetic, and carcinogenic damage. Likewise, Roy et al. [35] found that metronidazole’s genotoxicity may be due to the sensitization of bone marrow cells. In addition, Menendez et al. [36] indicated that metronidazole hydroxy metabolite, in rat hepatocytes, produced an increase in micronuclei and DNA breaks. As such, several available reports indicated that apoptosis or necrosis reflects DNA damage such as variation of bases, single-strand breaks, and crossing between DNA-protein or DNA-DNA, finally resulting in early embryonic defects [37]. It has been indicated that metronidazole also causes an increase in isochromatic and chromatid breaks [30]. Hence, we could establish that such degenerative damages and deleterious effects of metronidazole are implicated to a great extent in morphological defects and teratogenicity.

Indeed, intracellular metabolic conversion plays an important role in the cytotoxic activity of metronidazole. The reduced metronidazole binds to the DNA, enhancing the destabilization of helix strands and consequent DNA breakage [38]. Furthermore, the toxicity of metronidazole may result from its derivative, the thiamin analogue [39], or from free radical-mediated damage generated during metronidazole metabolism, which causes cell death [40]. The precise mechanism of action of metronidazole is unclear; however, the reduced form of metronidazole and free radicals can interact with DNA, leading to inhibition of DNA synthesis and DNA degradation, leading to cellular death [41].

Whatever the disruptor, an in utero placental lesion may cause prenatal growth retardation, early pregnancy loss, and increase the risk for fetal disorders through the placenta–organ axis [42]. In the present results, metronidazole produced placental hypotrophy associated with a decrease in placental weight and a reduction in the placental basal zone compared with the control. The collective findings from the present study suggest that the intrauterine fetal growth disorders and pronounced teratogenicity may be due to the disruption of normal placental morphology due to the toxic effects of metronidazole after metabolic reduction. In addition, defects in placental architecture may include the histopathology of placental zones. These also include placental blood disorders, ensuring that metronidazole implicates angiogenesis during early placental development [43]. 

Indeed, intrauterine embryonic lethality or viability emerges from labyrinth defects as a prominent interface of placental disorders. Many studies supported the idea that there is a direct relationship between the labyrinth and fetal development [44]. Micropathologically, the decrease in placental weight observed in the present finding refers to the apoptosis, necrosis, and degeneration of trophoblasts due to placental damage induced by metronidazole [45]. Furthermore, the placenta discoloration, adhesion of the yolk sac on the chorionic surface of the placenta, and reduction in the labyrinth zone noticed macroscopically in the present findings might be due to placental necrosis in the trophoblasts of the labyrinth zone [46]. In the present results, there is an increase in intrauterine growth restriction (IUGR), indicating placental apoptosis that may be due to the mutagenic activity of metronidazole [47]. DNA damage, arrest of the cell cycle in trophoblasts, and diminished spongiotrophoblast proliferation may interpret the reduction in diameter in the labyrinth zone and basal zone, which is consistent with metronidazole cytotoxicity [48]. Furthermore, we can explain that a reduction in placenta size and placenta weight is attributed to the growth suppression of the labyrinth zone, the retardation of the development of the basal zone, and the cystic deterioration of glycogen cells induced by metronidazole [49].

The liver coefficients of pregnant rats in the GIII experimental group were significantly reduced. It indicates that the liver is one of the target organs for metronidazole, so the practical clinical significance needs further investigation in combination with blood biochemical indices and pathological sections.

After gavage, metronidazole is digested and absorbed by the gastrointestinal tract, metabolized in the liver, and the metabolites (hydroxy metronidazole) are excreted through the kidneys. The serum biochemical findings can further detect liver damage [50]. Elevations in the levels of ALP, ALT, AST, and total protein are a sign of liver damage. The levels of total protein can reflect protein synthesis ability and immunity [51]. The liver is an important organ for metabolism and is the main site of fat and protein metabolism [52]. TC, TG, LDL-C, and HDL-C indicators are associated with dyslipidemia [53]. Therefore, biochemical parameters are determined in the present study using data obtained from the liver. Based on the present findings, the GIII group increased the levels of ALT, AST, and ALP, and the changes were more evident with an increase in the experimental period (0–20th dpc). In general, increases in serum concentration levels of ALT, AST, ALP, and TP are biological markers of hepatic damage [54]. The effect on the liver, a pivotal organ of metabolic homeostasis, is reflected in the levels of AST [55]. Total protein is one of the important indicators of biochemical detection that plays an immune role during the administration of toxic substances (xenobiotics). Therefore, the simultaneous elevation in TP levels often indicates the presence of toxicity in the body [56]. This suggests that metronidazole may cause hepatocellular damage and abnormal liver metabolic function. The GIII caused elevations of hepatic function indicators, and their elevation often indicates excessive protein intake or abnormal hepatic metabolism [57]. Studies have reported that liver dysfunction often leads to disturbances in lipid metabolism, resulting in increased serum TG [58]. The same results were observed in the GIII; thus, it was assumed that metronidazole may have an increased risk of liver dysfunction and abnormal lipid metabolism. 

## 8. Conclusions

The developmental defects observed in present findings disclose the potency of metronidazole administration on pregnancy outcomes and have pathological effects on the placental development of pregnant female rats. Furthermore, the toxic effects of metronidazole are evidenced by a significant intrauterine fetal growth restriction and teratogenicity. In addition, metronidazole causes significant impacts on the maternal liver in pregnant rats at GIII and affects their lipid metabolism. The toxicity was also extended to the fetal liver, mainly by the maternal-fetal-placental vectors. Compared with the control group, all experimental groups showed varying degrees of histopathological alterations, including hepatocyte damage and increased inflammatory cells. Hepatic histopathology showed that the GIII group had the most severe liver tissue damage compared with the GI and GII groups. These findings were consistent with the biochemical index findings. So, from the presently established findings, we can conclude that metronidazole administration is unsafe during gestation for dams and fetuses and should be strictly advised and prescribed for its use and prescription. Additionally, further consideration should be given to the associated health risks.

## Figures and Tables

**Table 1:** Pregnancy outcomes of female rats treated with metronidazole at different gestation periods and sacrificed on the 20th day of gestation.

Parameters	Experimental Groups			
	GC	GMI	GMII	GMIII
Number of litters	10	10	10	10
Number of fetuses	132.3	77.80	68.20	60.80
Number of implantation sites/litter	13.50 ± 1.25	8.75 ± 0.95 *	8.69 ± 0.85 *	7.28 ± 0.95 *
Number of resorption sites/litter	0.27 ± 0.015	0.97 ± 0.013 *	1.21 ± 0.002 *	1.42 ± 0.002 *
Number of dead fetuses/litter	0.00	0.00	0.66 ± 0.04 *	0.78 ± 0.04 *
Number of live fetuses/litter	13.23 ± 0.57	7.78 ± 1.30 *	6.82 ± 1.30 *	6.08 ± 1.30 *
Fetal body weight (g)	5.02 ± 0.130	4.89 ± 0.300	3.52 ± 0.167 *	2.99 ± 0.090 *
Crown-Rump Length (mm)	40.01 ± 1.42	39.20 ± 2.21	26.32 ± 1.12 *	21.50 ± 1.23 *

Values are expressed as mean ± standard deviation (mean ±SD) *n*= 10/group. * The values are significantly different at *p* ≤ 0.05 (ANOVA) with Duncan’s multiple range test.

**Table 2:** Effect of metronidazole on lipid profile in pregnant albino rats.

Lipid Index	Groups			
	GC	GMI	GMII	GMIII
TC (mg/dL)	81.51 ± 5.11	82.61 ± 6.12	85.43 ± 4.22	131.42 ± 7.12 *
TG (mg/dL)	89.14 ± 6.31	88.33 ± 5.67	87.86 ± 5.17	166.8 ± 10.31 *
HDL (mg/dL)	37.33 ± 3.22	40.21 ± 4.01	42.62 ± 3.15	35.04 ± 2.09
LDL (mg/dL)	16.24 ± 3.46	17.88 ± 1.96	18.21 ± 1.77	31.22 ± 2.41 *

Values are expressed as the mean ± standard deviation (mean ± SD). (*n* = 10). * Significant difference with respect to the control group at *p* ≤ 0.05 (ANOVA) with Duncan’s multiple range test.

## Data Availability

Data is contained within the article.
